# Lipoprotein profiling in early multiple sclerosis patients: effect of chronic inflammation?

**DOI:** 10.1186/s12944-020-01221-x

**Published:** 2020-03-17

**Authors:** Žofia Rádiková, Adela Penesová, Miroslav Vlček, Andrea Havranová, Monika Siváková, Pavel Šiarnik, Ingrid Žitňanová, Richard Imrich, Peter Turčáni, Branislav Kollár

**Affiliations:** 1 Institute of Clinical and Translational Research, Biomedical Research Center, Slovak Academy of Sciences, Dúbravská cesta 9, SK 845 05 Bratislava, Slovakia; 2 1st Department of Neurology, Faculty of Medicine, Comenius University, Bratislava, Slovakia; 3 Institute of Medical Chemistry, Biochemistry and Clinical Biochemistry, Faculty of Medicine, Comenius University, Bratislava, Slovakia

**Keywords:** Multiple sclerosis, Inflammation, Cytokines, Lipoproteins, HDL cholesterol subfractions

## Abstract

**Background:**

Inflammatory cytokines contribute to proatherogenic changes in lipid metabolism by reduction of HDL-cholesterol (HDL-C) levels, impairment of its antiinflammatory and antioxidant functions. Therefore, the protective actions of HDL-C can be limited in chronic inflammatory diseases such as multiple sclerosis (MS). The aim of this study was to assess the association between lipoprotein subfractions and inflammatory status in early stages of multiple sclerosis.

**Methods:**

Polyacrylamide gel electrophoresis Lipoprint© System was used for lipoprotein profile analysis in 19 newly diagnosed MS patients, and in matched 19 healthy controls. Serum levels of interleukin (IL) 1β, IL-2, IL-4, IL-5, IL-6, IL-7, IL-8, IL-10, IL-12 (p70), IL-13, IL-17, granulocyte colony-stimulating factor (G-CSF), granulocyte-macrophage colony-stimulating factor, interferon-γ and TNF-α were measured by multiplex bead assay.

**Results:**

Concentrations of the measured cytokines and lipoprotein subclasses were comparable between MS patients and controls. Male, but not female MS patients had significantly higher total HDL-C and small HDL-C subfraction than healthy controls. Large HDL-C negatively correlated with all measured cytokines except IL-17 in MS but not in controls. Intermediate HDL-C subfractions correlated positively with all measured cytokines except G-CSF in MS females but not in MS males or controls.

**Conclusion:**

Our results of higher HDL-C and mainly its small HDL-C subfraction suggest that male MS patients are at higher risk of atherosclerosis and the subtle dyslipidemia is present in early stages of the disease. The correlations between specific HDL-C subfractions and the inflammatory cytokines demonstrate mutual links between systemic inflammation and lipid metabolism in MS.

**Trial registration:**

ClinicalTrials.gov, Identifier: NCT 03052595 Registered on Feb 14, 2017.

## Introduction

Multiple sclerosis (MS) is a chronic neuroinflammatory disease of the central nervous system (CNS), leading to demyelination and neurodegeneration. The most frequent symptoms include motor impairment, visual disturbances, sensory problems, pain, fatigue and cognitive impairment, leading to serious physical disabilities in young adults [1].

MS patients have increased cardiovascular risk even in the absence of traditional risk factors such as obesity, hypertension, type 2 diabetes or dyslipidemia suggesting disease-related factors contribute to the development of atherosclerosis in MS [2–5].

The relationship of dyslipidemia, namely of elevated total cholesterol, low density lipoprotein cholesterol (LDL-C) and decreased high density lipoprotein cholesterol (HDL-C) levels, to atherogenesis and cardiovascular diseases is well accepted ([6]; NCEP and ATP III, 2002). LDL-C and HDL-C represent a heterogeneous group of particles that differ in density, migration characteristics, apoprotein content and relationships to disease and these subfractions vary in their risk profile. In particular, small dense LDL-C particles are associated with increased cardiovascular risk, metabolic syndrome and type 2 diabetes, while large LDL-C subfractions were not found to be associated with cardiovascular risk [7, 8]. Although the role of HDL-C subfractions in cardioprotective, anti-inflammatory and anti-oxidative mechanisms is less clear, the majority of studies consider large HDL-C subfractions more protective than the small HDL-C [6, 8–13].

Inflammatory cytokines can contribute to proatherogenic changes in lipid metabolism by alterations in the enzymes and apolipoproteins associated with HDL-C. This leads to a reduction of HDL-C levels and to an impairment of anti-inflammatory, antioxidant, and reverse cholesterol transport functions [14, 15]. Thus, chronic inflammation may represent an important factor in the development of dyslipidemia, atherosclerosis, cardiovascular disease, metabolic syndrome and obesity in MS [16–20].

Furthermore, several studies showed links between serum cholesterol profiles and disease outcomes in MS [17, 19, 21, 22]. Recently, we showed decreased insulin sensitivity and increased insulin secretion in response to oral glucose load unrelated to inflammatory and physical activity status in early MS patients [23]. In addition, we reported a negative association of IDL-B lipoprotein subfraction with the parameters of insulin resistance and hyperinsulinemia suggesting an incipient dyslipidemia preceded by insulin resistance development in these MS patients [24].

It remains unclear to what extend dyslipidemia is associated with inflammation in early stages of the disease. Our study was aimed at (1) evaluation of dyslipidemia and (2) exploration of the relationship between specific lipoprotein subfractions and inflammatory status in patients with early MS.

## Subjects and methods

### Participants and study protocol

A total of 19 (*n* = 19) patients (aged 20–45 years) with MS fulfilling the 2010 McDonald’s criteria after the first episode of symptoms and with an Expanded Disability Status Scale (EDSS) score ≤ 2.0, were recruited from the registry of the 1^st^ Department of Neurology, Medical Faculty, Comenius University, Bratislava, Slovakia. Nineteen (*n* = 19) age, sex and BMI matched healthy subjects served as controls. All the subjects studied (patients and controls) were non-smokers, had a negative history of cardiovascular, metabolic, endocrine, renal or hepatic disease, malignancy, acute or chronic infection or any current medication. The examinations of MS patients were performed at least 2 months after the pulse glucocorticoid treatment (methylprednisolone 1000 mg per day intravenously for 3–5 days) following the first MS episode. All MS patients were in remission and without any medication at the time of investigations. Clinical characteristics of the groups are shown in Table 1. The study was carried out according to the ethical standards of the National Research Committee and to the 1964 Helsinki declaration and its later amendments or comparable ethical standards. The study was approved by the Ethics Committee of the Faculty of Medicine, Comenius University and University Hospital in Bratislava as well as by the Ethics Committee of the Bratislava Self-Governing Region, Bratislava, Slovakia. After detailed explanation, all patients and controls signed informed consent before participating in the study.

**Table 1:** Clinical characteristics of MS patients and healthy subjects. Data are expressed as mean ± SD, in case of non-parametric distribution as median (25th - 75th percentile)

	MS (*n* = 19)	Controls (*n* = 19)
Men/women	9/10	9/10
Age (years)	30.4 ± 7.0	28.7 ± 6.7
BMI (kg/m^2^)	23.7 ± 4.5	22.2 (21.5–27.5)
EDSS (arbitrary units)	1.0 (1.0–1.5)	–
Body fat percentage (%)	27.1 ± 7.7	27.8 ± 7.6
Lean body mass (kg)	49.9 (41.9–62.7)	51.2 (42.3–63.8)
SBP (mmHg)	111 (107–115)	114 (110–132)
DBP (mmHg)	70 ± 8	73 ± 9
Heart rate (1/min)	67 ± 11	70 ± 10
hsCRP (mg/l)	0.63 (0.46–1.03)	0.80 (0.60–2.78)
TG (mmol/l)	0.67 (0.57–1.10)	0.73 (0.63–1.20)
Total cholesterol (mmol/l)	4.56 ± 0.93	4.08 ± 0.72
HDL cholesterol (mmol/l)	1.48 ± 0.35	1.31 (0.96–1.79)
LDL cholesterol (mmol/l)	2.45 ± 0.79	2.34 ± 0.61
METh/day	37 ± 6	40 ± 6
EE (kcal/24 h)	2707 ± 708	2986 ± 628

After an overnight fast, peripheral venous blood samples were collected into polyethylene tubes; after clotting at room temperature for about 60 min, the blood was centrifuged at 4 °C and aliquots of sera were stored at − 70 °C until assayed.

To assess the level of physical activity, the subjects completed the Slovak version of the Lagerros Energy Expenditure Questionnaire (EEQ) [25], to quantify the total energy output associated with all physical activity during an average week day. Physical activity was graded into nine steps according to its intensity, representing a multiple of metabolic energy turnover (MET). One MET represents an energy expenditure of 1 kcal/kg body weight per hour [25]. Participants reported the time spent on each intensity level during a typical day and night, total physical activity score and energy expenditure were calculated as the sum of the individual level activities (MET1*t1 + MET2*t2 + ... + MET9*t9 where METi represents the MET value for current level and it represents the time spent performing the activity) [25].

### Assays

Fasting serum total cholesterol (TC), LDL-C, HDL-C, and triglyceride (TG) levels were determined using an autoanalyzer (Siemens Healthcare Diagnostics Inc., Tarrytown, NY, USA) by standard procedures with enzymatic kits (Roche Diagnostics, Lewes, UK). Lipoprotein subfraction analysis was performed using high resolution polyacrylamide gel electrophoresis technique - Lipoprint system (Quantimetrix Corporation, Redondo Beach, CA, USA) according to the manufacturer’s manual, which enables the analysis of following lipoprotein subfractions profile: the very low-density lipoprotein (VLDL) fraction, the intermediate-density lipoprotein (IDL) C, B and A, the low-density lipoprotein (LDL) with subfractions 1 and 2 (large LDL) and subfractions 3 to 7 (small dense LDL - sdLDL), and the high density lipoprotein (HDL) subfractions categorized into large (subfractions 1–3), intermediate (subfractions 4–7), and small HDL (subfractions 8–10).

Levels of cytokines in serum were measured by multiplex bead assay (Bio-Plex Human Cytokine panel; Bio-Rad, Hercules, CA, USA). Supernatants were analyzed simultaneously for the following selected 15 cytokines: IL-1β, IL-2, IL-4, IL-5, IL-6, IL-7, IL-8, IL-10, IL-12 (p70), IL-13, IL-17, granulocyte colony-stimulating factor (G-CSF), granulocyte-macrophage colony-stimulating factor (GM-CSF), interferon-γ (IFN-γ), and TNF-α. Serum hsCRP was measured by immunoturbidimetric assay on automated biochemistry analyzer (Hitachi 917, Roche Diagnostics, Basel, Switzerland).

### Statistical evaluation

Statistical analysis of the obtained data was performed using the IBM SPSS Statistics version 19 (SPSS Inc., Chicago, IL, USA). The normality of continuous variables was assessed by the Kolmogorov-Smirnov test. Normally distributed data were expressed as mean ± SD, while data not normally distributed were expressed as median (interquartile range [IQR]). Between groups’ comparisons of the continuous variables were evaluated by the Student’s t-test or Mann-Whitney U test, as appropriate. Two approaches were used to examine the association of lipoprotein subfractions and inflammatory status. First, the correlations between various lipoprotein subfractions and cytokines measured were examined in MS patients’ and controls’ groups using Pearson’s or Spearman’s correlation coefficient, depending on the normality of data. The correlations were then performed when controlling for age, gender and BMI as well. Second, the correlations and the correlations when controlling for age and BMI were performed also in gender subgroups of patients and controls. Differences were considered significant at p<0.05.

## Results

**Fig. 1:**
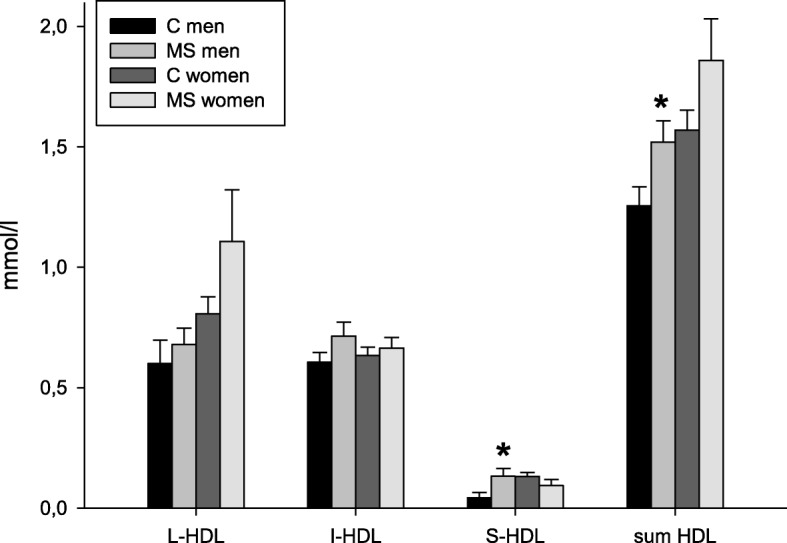
Concentrations of HDL subfractions in male and female subgroups of controls (C) and MS patients. L-HDL = large HDL cholesterol; I-HDL = intermediate HDL cholesterol; S-HDL = small HDL cholesterol; sum HDL = sum of large, intermediate and small HDL cholesterol. Data are expressed as means ± SEM, * *p* < 0.05 vs. control of the same gender

Anthropometric parameters and level of physical activity were comparable in both, the MS patients and healthy controls group (Table 1), even after comparison of male and female subgroup separately. There were no significant differences between study groups in fasting serum concentration of TG, and total, LDL-C, HDL-C as well as in their respective subfractions (Table 2). When analyzed by gender, there was no significant difference in lipid parameters between female patients and female controls. Male patients had significantly higher HDL-C concentrations (MS-M: 1.28 ± 0.23 mmol/l vs. C-M: 1.04 ± 0.23 mmol/l; *p* = 0.037). The similar trend was observed in small HDL-C subfraction (MS-M: 4.0 (2.5–7.0) mg/dl vs. C-M: 1.0 (0.0–2.0) mg/dl; *p* = 0.015) (Fig. 1). The physical activity (expressed as calculated energy expenditure in kcal/24 h) was comparable in men with MS and in controls (MS-M: 3055 ± 784 kcal/24 h vs. C-M: 3383 ± 620 kcal/24 h; *p* = 0.366). The concentrations of 15 measured cytokines were not significantly different between the patients and controls (Table 3), not even after within-gender comparison.

**Table 2:** Concentrations of the cholesterol subfractions measured by Lipoprint in MS patients and controls. Data are expressed as mean ± SD, in case of non-parametric distribution as median (25th - 75th percentile)

	MS (*n* = 19)	Controls (*n* = 19)
VLDL (mmol/l)	0.757 ± 0.328	0.595 (0.524–0.847)
IDL-A (mmol/l)	0.218 ± 0.105	0.216 ± 0.086
IDL-B (mmol/l)	0.259 (0.207–0.310)	0.233 (0.181–0.284)
IDL-C (mmol/l)	0.885 ± 0.243	0.834 ± 0.198
LDL1 (mmol/l)	0.361 ± 0.273	0.404 ± 0.230
LDL2 (mmol/l)	0.207 (0.000–0.259)	0.200 ± 0.217
LDL3–7 (mmol/l)	0.000 (0.000–0.052)	0.000 (0.000–0.039)
Large HDL (mmol/l)	0.763 (0.595–0.957)	0.709 ± 0.271
Intermediate HDL (mmol/l)	0.646 (0.543–0.802)	0.621 ± 0.113
Small HDL (mmol/l)	0.111 ± 0.085	0.090 ± 0.072
Total cholesterol (mmol/l)	4.54 ± 0.95	4.07 ± 0.73

**Table 3:** Concentrations of selected cytokines. Data are expressed as mean ± SD, in case of non-parametric distribution, median (25th - 75th percentile)

	MS (*n* = 19)	Controls (*n* = 19)
IL-1β (pg/ml)	2.48 ± 1.08	2.23 ± 0.97
IL-2 (pg/ml)	10.55 ± 7.56	9.73 ± 7.07
IL-4 (pg/ml)	4.58 ± 1.94	4.06 ± 2.40
IL-5 (pg/ml)	9.84 ± 6.13	8.20 ± 6.19
IL-6 (pg/ml)	9.51 ± 4.80	9.10 ± 6.10
IL-7 (pg/ml)	15.28 ± 8.16	14.07 ± 8.44
IL-8 (pg/ml)	30.99 ± 12.92	25.76 ± 14.43
IL-10 (pg/ml)	6.00 ± 3.52	5.67 ± 3.06
IL-12 (pg/ml)	18.63 ± 12.95	16.33 ± 11.69
IL-13 (pg/ml)	15.97 ± 6.77	16.59 ± 9.48
IL-17 (pg/ml)	29.06 (24.05–39.17)	30.30 ± 23.59
G-CSF (pg/ml)	134.7 ± 50.5	144.0 ± 79.1
GM-CSF (pg/ml)	23.29 ± 19.75	25.97 ± 21.57
IFN-γ (pg/ml)	302 ± 156	252 ± 167
TNF-α (pg/ml)	56.89 ± 41.18	51.53 ± 38.27

IL-7 negatively correlated with intermediate HDL-C and total HDL-C in healthy controls. GM-CSF negatively correlated with VLDL-C, IDL-C, large HDL-C and total HDL-C in healthy controls, when controlling for age, BMI and gender, the most robust were correlations of GM-CSF with large HDL-C subfraction and total HDL-C in healthy controls (Table 4). After data analysis by gender, male controls showed robust positive correlation of LDL1, LDL2, and LDL-C 1–2 subfractions with almost all cytokines (r = .700–0.992; *p* = 0.040–0.000). However, since all the LDL-C subfractions’ parameters in male controls correlated positively with BMI (r = 0.717–0.889; *p* = 0.03–0.000), data after controlling for age and BMI followed similar pattern like in the whole control group (Table 5). The strong negative correlations of GM-CSF were present only in female controls.

**Table 4:** Selected cytokines and their correlations with cholesterol subfractions in healthy controls after controlling for age, BMI and gender (*p < 0.05, **p < 0.01, and ***p < 0.001)

Controls	IL-1β	IL-2	IL-4	IL-5	IL-6	IL-7	IL-8	IL-10	IL-12	IL-13	IL-17	G-CSF	GM-CSF	IFN-γ	TNF-α
**VLDL (mg/dl)**	NS	NS	NS	NS	NS	NS	NS	NS	NS	NS	NS	NS	−0.571 *	NS	NS
**IDL-A (mg/dl)**	NS	NS	NS	NS	NS	NS	NS	NS	NS	NS	NS	NS	NS	NS	NS
**IDL-B (mg/dl)**	NS	NS	NS	NS	NS	NS	NS	NS	NS	NS	NS	NS	NS	NS	NS
**IDL-C (mg/dl)**	NS	NS	NS	NS	NS	NS	NS	NS	NS	NS	NS	NS	−0.715 **	NS	NS
**LDL1 (mg/dl)**	NS	NS	NS	NS	NS	NS	NS	NS	NS	NS	NS	NS	NS	NS	NS
**LDL2 (mg/dl)**	NS	NS	NS	NS	NS	NS	NS	NS	NS	NS	NS	NS	NS	NS	NS
**LDL1–2 (mg/dl)**	NS	NS	NS	NS	NS	NS	NS	NS	NS	NS	NS	NS	NS	NS	NS
**LDL3–7 (mg/dl)**	NS	NS	NS	NS	NS	NS	NS	NS	NS	NS	NS	NS	NS	NS	NS
**L-HDL (mg/dl)**	NS	NS	NS	NS	NS	NS	NS	NS	NS	NS	NS	NS	−0.819***	NS	NS
**I-HDL (mg/dl)**	NS	NS	NS	NS	NS	−0.545 *	NS	NS	NS	NS	NS	NS	NS	NS	NS
**S-HDL (mg/dl)**	NS	NS	NS	NS	NS	NS	NS	NS	NS	NS	NS	NS	NS	NS	NS
**Total HDL (mg/dl)**	NS	NS	NS	NS	NS	−0.582 *	NS	NS	NS	NS	NS	−0.570 *	−0.866***	NS	NS
**Total cholesterol (mg/dl)**	NS	NS	NS	NS	NS	NS	NS	NS	NS	NS	NS	NS	−0.659 **	NS	NS

**Table 5:** Correlation of selected cytokines with cholesterol subfractions in male and female subgroups of healthy controls after controlling for age and BMI (*p < 0.05, **p < 0.01, and ***p < 0.001)

Controls		IL-1β	IL-2	IL-4	IL-5	IL-6	IL-7	IL-8	IL-10	IL-12	IL-13	IL-17	G-CSF	GM-CSF	IFN-γ	TNF-α
**VLDL (mg/dl)**	**M**	NS	NS	NS	NS	NS	NS	NS	NS	NS	NS	NS	NS	NS	NS	NS
	**F**	NS	NS	NS	NS	NS	NS	NS	NS	NS	NS	NS	NS	−0.710 *	NS	NS
**IDL-A (mg/dl)**	**M**	NS	NS	NS	NS	NS	NS	NS	NS	NS	NS	NS	NS	NS	NS	NS
	**F**	NS	NS	NS	NS	NS	−0.800 *	NS	NS	NS	NS	NS	− 0.790 *	NS	NS	NS
**IDL-B (mg/dl)**	**M**	−0.928 **	NS	NS	NS	NS	NS	NS	NS	NS	NS	NS	NS	NS	NS	NS
	**F**	NS	NS	NS	NS	NS	NS	NS	NS	NS	NS	NS	NS	NS	NS	NS
**IDL-C (mg/dl)**	**M**	NS	NS	NS	NS	NS	NS	NS	NS	NS	NS	NS	NS	NS	NS	NS
	**F**	NS	NS	NS	NS	NS	NS	NS	NS	NS	NS	NS	NS	−0.791 *	NS	NS
**LDL1 (mg/dl)**	**M**	NS	NS	NS	NS	NS	NS	0.857 *	NS	NS	NS	NS	NS	NS	NS	NS
	**F**	NS	NS	NS	NS	NS	−0.717 *	NS	NS	NS	NS	NS	NS	NS	NS	NS
**LDL2 (mg/dl)**	**M**	NS	NS	NS	NS	NS	NS	NS	NS	NS	NS	NS	NS	NS	NS	NS
	**F**	NS	NS	NS	NS	NS	NS	NS	NS	NS	NS	NS	NS	NS	NS	NS
**LDL1–2 (mg/dl)**	**M**	NS	NS	NS	NS	NS	NS	NS	NS	NS	NS	NS	NS	NS	NS	NS
	**F**	NS	NS	NS	NS	NS	−0.719 *	NS	NS	NS	NS	NS	NS	NS	NS	NS
**LDL3–7 (mg/dl)**	**M**	NS	NS	NS	NS	NS	NS	NS	NS	NS	NS	NS	NS	NS	NS	NS
	**F**	NS	NS	NS	NS	NS	NS	NS	NS	NS	NS	NS	NS	NS	NS	NS
**L-HDL (mg/dl)**	**M**	NS	NS	NS	NS	NS	NS	0.823 *	NS	NS	NS	NS	NS	NS	NS	NS
	**F**	NS	NS	NS	NS	NS	NS	NS	NS	NS	NS	NS	NS	−0.948***	NS	NS
**I-HDL (mg/dl)**	**M**	NS	NS	−0.872 *	NS	NS	NS	NS	NS	NS	NS	NS	NS	NS	−0.875 *	− 0.821 *
	**F**	NS	NS	NS	NS	NS	NS	NS	NS	NS	NS	NS	NS	NS	NS	NS
**S-HDL (mg/dl)**	**M**	NS	NS	NS	NS	NS	NS	NS	NS	NS	NS	NS	NS	NS	NS	NS
	**F**	NS	NS	NS	NS	NS	NS	NS	NS	NS	NS	NS	NS	NS	NS	NS
**Total HDL (mg/dl)**	**M**	NS	NS	NS	NS	NS	NS	NS	NS	NS	NS	NS	NS	−0.758 *	NS	NS
	**F**	NS	NS	NS	NS	NS	−0.711 *	NS	NS	NS	NS	NS	−0.787 *	−0.940***	NS	NS
**Total cholesterol (mg/dl)**	**M**	NS	NS	NS	NS	NS	NS	NS	NS	NS	NS	NS	NS	NS	NS	NS
	**F**	NS	NS	NS	NS	NS	NS	NS	NS	NS	NS	NS	−0.756 *	−0.844 **	NS	NS

In patients with MS the situation was different (Table 6). In spite of the fact, that the total cholesterol did not correlate with any of the cytokine levels, the individual lipoprotein subfractions showed several significant correlations. None of the LDL-C subfractions correlates with cytokine levels when analyzing all patients controlled for age and BMI (Table 6). After within-gender analyses of the MS patients, few positive correlations appeared in the male subgroup of MS patients regarding the large LDL-C 1, 2, 1–2 subfractions with IL-4, IL-6, IL-12, and TNFα (Table 7). Surprisingly, small dense LDL-C subfractions did not show any relationship to cytokine levels, neither in MS patients *en bloc*, nor in separate gender subgroups after controlling for age and BMI. On the other hand, in MS patients predominantly the large HDL-C subfraction and by some extent total HDL-C and intermediate HDL-C subfractions correlated with the cytokine levels. Both, total HDL-C and large HDL-C subfractions correlated negatively with inflammatory cytokines, whereas intermediated HDL-C subfractions showed positive correlations (Table 6). This trend was even more pronounced in female MS patients, but not in male MS patients (Table 7). In female MS patients, the large HDL-C subfractions showed a significant negative correlation with majority of the cytokines with an r value in the range from − 0.712 to − 0.882 (*p* = 0.048–0.004). In contrast, intermediate HDL-C subfractions correlated positively with almost all cytokine levels (*r* = 0.718–0.887, *p* = 0.045–0.003) in female MS patients when controlled for age and BMI (Table 6), however no significant correlation was present in the male subgroup of MS patients; with no significant correlation in the small HDL-C subfraction for both genders (Table 7).

**Table 6:** Selected cytokines and their correlations with cholesterol subfractions in MS patients after controlling for age, BMI and gender (*p < 0.05, **p < 0.01)

MS patients	IL-1β	IL-2	IL-4	IL-5	IL-6	IL-7	IL-8	IL-10	IL-12	IL-13	IL-17	G-CSF	GM-CSF	IFN-γ	TNF-α
**VLDL (mg/dl)**	NS	NS	NS	NS	NS	NS	NS	NS	NS	NS	NS	NS	− 0.655 **	NS	NS
**IDL-A (mg/dl)**	NS	NS	NS	NS	NS	NS	NS	NS	NS	NS	NS	NS	0.739 **	NS	NS
**IDL-B (mg/dl)**	NS	NS	NS	NS	NS	NS	NS	NS	NS	NS	NS	NS	0.684 **	NS	NS
**IDL-C (mg/dl)**	NS	NS	NS	NS	NS	NS	NS	NS	NS	NS	NS	NS	NS	NS	NS
**LDL1 (mg/dl)**	NS	NS	NS	NS	NS	NS	NS	NS	NS	NS	NS	NS	NS	NS	NS
**LDL2 (mg/dl)**	NS	NS	NS	NS	NS	NS	NS	NS	NS	NS	NS	NS	NS	NS	NS
**LDL1–2 (mg/dl)**	NS	NS	NS	NS	NS	NS	NS	NS	NS	NS	NS	NS	NS	NS	NS
**LDL3–7 (mg/dl)**	NS	NS	NS	NS	NS	NS	NS	NS	NS	NS	NS	NS	NS	NS	NS
**L-HDL (mg/dl)**	−0.600 *	−0.577 *	−0.579 *	−0.640 **	−0.711 **	−0.625 *	−0.691 **	−0.626 *	−0.649 **	−0.558 *	NS	−0.587 *	−0.685 **	−0.614 *	− 0.589 *
**I-HDL (mg/dl)**	NS	NS	NS	0.541 *	NS	NS	NS	NS	0.602 *	0.553 *	NS	NS	NS	NS	0.561 *
**S-HDL (mg/dl)**	NS	NS	NS	NS	NS	NS	NS	NS	NS	NS	NS	NS	NS	NS	NS
**Total HDL (mg/dl)**	NS	NS	NS	NS	−0.589 *	NS	−0.564 *	−0.527 *	NS	NS	NS	−0.551 *	− 0.602 *	NS	NS
**Total cholesterol (mg/dl)**	NS	NS	NS	NS	NS	NS	NS	NS	NS	NS	NS	NS	NS	NS	NS

**Table 7:** Correlation of selected cytokines with cholesterol subfractions in male and female subgroups of MS patients after controlling for age and BMI (*p < 0.05, **p < 0.01)

Controls		IL-1β	IL-2	IL-4	IL-5	IL-6	IL-7	IL-8	IL-10	IL-12	IL-13	IL-17	G-CSF	GM-CSF	IFN-γ	TNF-α
**VLDL (mg/dl)**	**M**	NS	NS	NS	NS	NS	NS	NS	NS	NS	NS	NS	NS	NS	NS	NS
	**F**	NS	NS	NS	NS	NS	NS	NS	NS	NS	NS	NS	NS	−0.797 *	NS	NS
**IDL-A (mg/dl)**	**M**	NS	NS	NS	NS	NS	NS	NS	NS	NS	NS	NS	NS	0.950 **	NS	NS
	**F**	NS	NS	NS	NS	NS	NS	NS	NS	NS	NS	NS	NS	NS	NS	NS
**IDL-B (mg/dl)**	**M**	NS	NS	NS	NS	NS	NS	NS	NS	NS	NS	NS	NS	0.884 *	NS	NS
	**F**	0.738 *	0.793 *	NS	NS	NS	NS	NS	NS	0.773 *	NS	0.721 *	NS	NS	NS	NS
**IDL-C (mg/dl)**	**M**	NS	NS	NS	NS	NS	NS	NS	NS	NS	NS	NS	NS	NS	NS	NS
	**F**	NS	NS	NS	NS	NS	NS	NS	NS	NS	NS	NS	NS	NS	NS	NS
**LDL1 (mg/dl)**	**M**	NS	NS	0.818 *	NS	NS	NS	NS	NS	NS	NS	NS	NS	NS	NS	NS
	**F**	NS	NS	NS	NS	NS	NS	NS	NS	NS	NS	NS	NS	NS	NS	NS
**LDL2 (mg/dl)**	**M**	NS	NS	NS	NS	0.869 *	NS	NS	NS	0.816 *	NS	NS	NS	NS	NS	0.847 *
	**F**	NS	NS	NS	NS	NS	NS	NS	NS	NS	NS	NS	NS	NS	NS	NS
**LDL1–2 (mg/dl)**	**M**	NS	NS	NS	NS	0.880 *	NS	NS	NS	0.811 *	NS	NS	NS	NS	NS	NS
	**F**	NS	NS	NS	NS	NS	NS	NS	NS	NS	NS	NS	NS	NS	NS	NS
**LDL3–7 (mg/dl)**	**M**	NS	NS	NS	NS	NS	NS	NS	NS	NS	NS	NS	NS	NS	NS	NS
	**F**	NS	NS	NS	NS	NS	NS	NS	NS	NS	NS	NS	NS	NS	NS	NS
**L-HDL (mg/dl)**	**M**	NS	NS	NS	NS	NS	NS	NS	NS	NS	NS	NS	NS	NS	NS	NS
	**F**	NS	NS	−0.718 *	−0.712 *	−0.823 *	−0.722 *	−0.771 *	−0.807 *	NS	−0.745 *	NS	NS	−0.882 **	NS	−0.726 *
**I-HDL (mg/dl)**	**M**	NS	NS	NS	NS	NS	NS	NS	NS	NS	NS	NS	NS	NS	NS	NS
	**F**	0.878 **	0.865 **	0.765 *	0.826 *	0.718 *	0.732 *	0.817 *	0.788 *	0.887 **	0.753 *	0.845 **	NS	0.865 **	0.757 *	0.790 *
**S-HDL (mg/dl)**	**M**	NS	NS	NS	NS	NS	NS	NS	NS	NS	NS	NS	NS	NS	NS	NS
	**F**	NS	NS	NS	NS	NS	NS	NS	NS	NS	NS	NS	NS	NS	NS	NS
**Total HDL (mg/dl)**	**M**	NS	NS	NS	NS	NS	NS	NS	NS	NS	NS	NS	NS	NS	NS	NS
	**F**	NS	NS	NS	NS	−0.784 *	NS	NS	−0.744 *	NS	NS	NS	NS	−0.787 *	NS	NS
**Total cholesterol (mg/dl)**	**M**	NS	NS	0.773 *	NS	NS	NS	NS	NS	0.725 *	NS	NS	NS	NS	NS	NS
	**F**	NS	NS	NS	NS	NS	NS	NS	NS	NS	−0.722 *	NS	−0.731 *	NS	NS	NS

## Discussion

The aim of this study was to investigate dyslipidemia in early MS in the context of inflammatory status. Despite the low inflammatory activity and low disability score in our MS patients, our results show higher total HDL-C and higher small HDL-C subfraction in male MS subjects compared to healthy male individuals. These findings suggest the presence of subtle signs of dyslipidemia at the early stages of the disease and in the absence of other risk factors such low physical activity or obesity. Furthermore, a distinct pattern of correlations between HDL-C subfractions and inflammatory cytokines in MS confirms existence of mutual relationships between lipid metabolism and inflammation.

LDL-C and HDL-C are heterogeneous groups of particles; their subfractions differ in size, density, in lipid and apolipoprotein compositions. There are several methods to measure lipoprotein subfractions, besides the Lipoprint method used in this study, for example Nuclear Magnetic Resonance Spectroscopy, gradient gel electrophoresis, ultracentrifugation, Vertical Auto Profile, and one of the newest – Anion-Exchange High-Performance Liquid Chromatography etc. [11, 26, 27]. These different subfractions have different impact on the risk of cardiovascular diseases; while it is more or less established that small dense LDL have atherogenic potential [8, 10, 28], the role of specific HDL-C subfractions remains equivocal. It has been suggested, that the large HDL-C particles may be more atheroprotective than small HDL-C particles [10, 13]. These different risk potentials of the respective subfractions may not be apparent in the clinical setting [2]. Therefore, shifts in these subfractions may explain an increase in cardiovascular risk in subjects with normal routine lipid profile creating basis for personalized lifestyle modifications such as smoking cessation, exercise, reduced alcohol consumption and/or nutritional interventions [29, 30].

Compared to HDL and LDL cholesterol, there is only limited information on the IDL-C and its relationship to cardiovascular risk, the data on IDL-C subfractions and their association with MS or inflammation in general are even scarcer. IDL-C has proatherogenic properties representing a considerable portion of cardiovascular risk attributed to non-HDL-C [31, 32].

The main function of HDL-C molecules is the reverse cholesterol transport from peripheral tissues to the liver. Furthermore, HDL-C has several antiatherogenic activities beyond the lipid transport, including anti-inflammatory, immunomodulatory, vasodilatory, antiapoptotic, antithrombotic and antioxidant properties [6, 19, 33, 34]. However, there is growing evidence indicating that the antiatherogenic effects of HDL-C are impaired in inflammatory state; affected are predominantly the small HDL-C particles [6]. Palavra et al. [2] reported increased cardiovascular risk in MS patients due to the elevation of, among others, small HDL-C. In atherosclerosis, a shift from larger towards smaller HDL-C particles in the HDL-C profile was observed [35]. Interestingly, lean patients with relapsing-remitting MS showed increased concentrations of small HDL-C and increased lipoprotein insulin resistance index [36], which may promote the progression of disease in these patients. The lipoprotein insulin resistance index is an algorithm calculating insulin resistance from six lipoprotein measures (3 subclases and 3 particle sizes) obtained from NMR spectroscopy, associated with both hepatic and peripheral insulin resistance as well [37]. Interestingly, in our study, the increased levels of small HDL-C particles were observed only in male, but not in female MS patients. However, small HDL-C subfraction did not correlate with any cytokines neither in controls, nor in patients.

HDL-C possesses atheroprotective functions and anti-inflammatory properties, but with the onset of systemic inflammation, it may become pro-inflammatory [33]. The dysfunctional HDL-C particles are those, which lost their atheroprotective features and can even exhibit the pro-atherogenic ones [38]. The causes of this phenomenon can be explained by amyloidosis and other translational and posttranslational modifications of apolipoprotein A-1 (apoA-1) [34]. Apolipoprotein A-1 is the major protein component of HDL-C particles in plasma. Besides the replacement of apoA-1 with serum amyloid A protein [6, 34, 35], alterations of apoA-1 by myeloperoxidase (chlorination, oxidation, nitration, carbamylation) and by reactive carbonyls (oxidation, glycation) during systemic inflammation lead to the production of dysfunctional HDL-C and to the transformation of this originally anti-inflammatory molecule into a pro-inflammatory one [34]. Furthermore, autoantibodies to apoA-1 and HDL-C have been reported in patients with other autoimmune conditions such as systemic lupus erythematosus, rheumatoid arthritis and antiphospholipid syndrome with reported higher cardiovascular risk and may therefore represent another mechanism potentially leading to proinflammatory properties of apoA-1/HDL-C [34].

The currently accepted concept of HDL-C metabolism describes secretion of small HDL-C particles by the liver and intestine followed by maturation, remodeling and size increase in the circulation by uptake and esterification of cellular cholesterol [39–41]. However, recent studies doubted this HDL-C size expansion model of metabolism suggesting HDL-C metabolism occurs mainly within its secreted size rather than progressive maturation of growing particles [39, 40].

Data regarding HDL-C and MRI measures of lesional and neurodegenerative changes in patients with MS have been equivocal [17, 19, 21, 22], which may be explained by the fact that these studies did not differentiate between HDL-C subfractions [42].

The consistent negative correlation of both, total HDL-C and large HDL-C subfraction with the cytokines can be explained by the fact, that routine clinical measurement of plasma HDL-C primarily reflects the levels of large, cholesterol-rich HDL-C particles with a frequent insensitivity to detect small, cholesterol-poor HDL-C subfraction [6]. In our study, the increased concentrations of small HDL-C subfraction in male MS patients and the opposing correlations of large and intermediate HDL-C subfractions in female MS patients suggest interactions between HDL-C and inflammation, even in remission and low inflammatory activity.

Increased levels of small HDL-C in a group of MS patients were found previously [2]. Interestingly, those patients were predominantly women (77%) and were diagnosed with MS for at least 6 months, unlike our patients, which were examined within the first 6 months after the diagnosis of MS. In another study [36], lean patients of both genders with MS with a mean disease duration of 13 years had increased small HDL-C when compared to matched controls. In our study, significantly increased levels of total HDL-C and small HDL-C subfractions were found in male MS patients when compared to their healthy counterparts, but not in women. These findings cannot be explained by a favorable effect of physical activity [7, 43], since the male MS patients in our study had comparable, even slightly lower physical activity level than controls. Therefore, the indicated gender differences in small HDL-C subfraction as well as the presented opposing correlations of large HDL-C and intermediate HDL-C subfractions may suggest a difference in the HDL-C response to inflammation in individuals with higher (women) and lower (men) normal levels of HDL-C, or at least the gender related time-dependence of the development of observed changes since the onset of the disease. This is supported by the fact, that the negative correlation of large HDL-C subfraction with all cytokines was found only in female MS patients. The positive correlation of intermediate HDL-C subfractions with several cytokines, which is in contrast to the negative correlation of large HDL-C subfraction with the cytokines, could indicate the features of intermediate HDL-C subfraction being opposite to large HDL subfraction.

Granulocyte-macrophage colony-simulating factor (GM-CSF), originally identified as a hematopoietic growth factor has been recently identified as a prominent factor playing role in inflammation and autommunity [44, 45]. This cytokine apparently plays an important role in the pathogenesis of MS, especially in modulation of myeloid cell function and potentially direct triggering of tissue destruction by these cells [46]. In our study, GM-CSF showed the most robust correlations with the lipoprotein subfractions, confirming its supposed role in the pathogenesis of MS.

An important limitation of our study, particularly regarding the gender differences, is the sample size (only 19 patients were included and analyzed). However, this drawback is counterbalanced by careful diagnosis and selection of the patients and matched controls. Besides this, none of our patients and controls was treated with any lipid lowering drugs, excluding their possible and meaningful interference with our results. Although assessment of MS patients in remission allowed us to analyze potentially “long-term” effects of the disease, higher inflammatory activity would probably exert stronger effects on lipoprotein subfractions. It is also possible, that the selected cytokines analyzed do not represent the triggers of the inflammation in MS. Identifying more representative cytokines expects further investigations. One could object the high-dose pulse glucocorticoid treatment at the time of the diagnosis. Generally, the high-dose pulse glucocorticoid treatment was introduced into the therapy to increase the efficacy and decrease the complications of long-term treatment, among other the metabolic consequences. However, our examination was performed at the time, when the patients were free of any steroids (intravenous or oral) or any other form of medication for at least 2 months. This could be sufficient to avoid the effect of glucocorticoid on the lipid metabolism.

## Conclusions

In conclusion, our results show higher HDL-C and the small HDL-C subfraction in males with early MS suggesting that male MS patients might be at higher risk of atherosclerosis development. Our results also clearly demonstrate a presence of subtle dyslipidemia in early stages of the disease. The observed pattern of correlations between HDL-C subfractions and several cytokines reflect mutual links between systemic inflammation and lipid metabolism in early MS with low inflammatory activity.

## Data Availability

The datasets used and/or analysed during the current study are available from the corresponding author on reasonable request.

## Abbreviations

apoA-1: Apolipoprotein A-1
ATP III: Adult treatment panel III
BBB: Blood-brain-barrier
BMI: Body mass index
CNS: Central nervous system
DBP: Diastolic blood pressure
EDSS: Expanded disability status scale
G-CSF: Granulocyte colony-stimulating factor
GM-CSF: Granulocyte-macrophage colony-stimulating factor
HDL-C: High density lipoprotein cholesterol
hsCRP: High sensitivity C-reactive protein
IDL: Intermediate density lipoprotein
IFN-γ: Interferon gamma
IL: Interleukin
LDL-C: Low density lipoprotein cholesterol
MS: Multiple sclerosis
NCEP: National cholesterol education program
NMR: Nuclear magnetic resonance
SBP: Systolic blood pressure
TC: Total cholesterol
TG: Triglycerides
TNF-α: Tumor necrosis factor alpha
VLDL: Very low density lipoprotein
